# Inertial Sensor-Based Analysis of Cervical and Upper-Thoracic Motion During Extrication: SNAID^®^ vs. Rautek Maneuver

**DOI:** 10.3390/s26144394

**Published:** 2026-07-10

**Authors:** Antonio J. Segura-Fornieles, Verónica V. Márquez-Hernández, Alba García-Viola, Aarón-Raúl Poyatos-Bakker, Mᵃ Carmen Rodríguez-García, Alfredo Alcayde-García, José M. Garrido-Molina

**Affiliations:** 1 Regimiento Acorazado “Alcázar de Toledo” Nº 61, Brigada Guadarrama XII, 28760 Madrid, Spain; ajsegura94@gmail.com; 2 Research Group of Health Sciences CTS-1127, Department of Nursing, Physiotherapy and Medicine, Faculty of Health Sciences, Universidad de Almeria, 04120 Almería, Spain; mrg451@ual.es; 3 Distrito Sanitario Almería, 04009 Almería, Spain; albagarciaviola@hotmail.com; 4 CIEMAT-Plataforma Solar de Almería-CIESOL, 04200 Tabernas, Spain; aaron.pb@psa.es; 5 Department of Engineering, Universidad de Almería, 04120 Almería, Spain; aalcayde@ual.es; 6 Centro de Emergencias Sanitarias 061, Servicio Andaluz de Salud, 04009 Almería, Spain; jgarrido22@gmail.com

**Keywords:** biomechanics, cervical spine, emergency care, extrication, inertial measurement units, motion analysis, spinal immobilization, trauma

## Abstract

**Highlights:**

**What are the main findings?**

- The SNAID^®^ device significantly reduces cervical lateral flexion-extension and intersegmental head–trunk motion compared with the Rautek maneuver during simulated extrication.
- The Rautek maneuver generates substantially higher global and relative spinal motion, particularly in the shoulder and head–trunk complex, indicating greater biomechanical desynchronization.

**What are the implications of the main findings?**

- The reduced cervical motion observed with SNAID^®^ indicates greater restriction of cervical and intersegmental movement during simulated extrication.
- Differences in segmental coupling indicate that device-based restraint systems may modify biomechanical stability and movement coupling.

**Abstract:**

Background: Spinal immobilization during extrication is a key component of trauma care aimed at reducing secondary neurological injury, although conventional techniques such as the Rautek maneuver may induce unintended spinal motion that could increase biomechanical stress on vulnerable structures. Recent developments, including the SNAID^®^ cervical restraint system, have been designed to improve motion restriction while maintaining operational feasibility in emergency settings. This study aimed to quantitatively compare spinal kinematics during extrication using SNAID^®^ versus the Rautek maneuver in a simulated environment. Methods: A controlled experimental study was conducted with 15 nursing students performing standardized extrication tasks under both conditions. Four synchronized 9-axis inertial measurement units were placed at the occiput, C7, sternum, and left shoulder to capture kinematic data at 100 Hz. Range of motion (ROM) was calculated for absolute and intersegmental movements, and statistical comparisons were performed using paired non-parametric tests with effect size estimation. Results: The results showed that SNAID^®^ significantly reduced cervical lateral flexion-extension compared with Rautek (*p* = 0.013, r_m_^b^ = −0.71), as well as markedly reducing head–trunk relative motion, particularly in sagittal flexion-extension (*p* < 0.001, r_m_^b^ = −0.99). In contrast, Rautek produced significantly greater shoulder and trunk motion, with the largest effect observed in shoulder lateral displacement (*p* < 0.001, r_m_^b^ = −0.99). Conclusions: the SNAID^®^ system demonstrated reduced cervical and intersegmental motion compared with the Rautek maneuver under standardized simulation conditions. These findings indicate differences in biomechanical behavior between both approaches; however, their clinical significance cannot be established from the present study and should not be interpreted as evidence of improved patient outcomes or neurological protection.

## 1. Introduction

At the global level, road traffic crashes are estimated to cause the deaths of more than 3000 individuals per day, corresponding to approximately two fatalities per minute [1]. Beyond mortality, injuries resulting from these events constitute one of the leading causes of permanent disability worldwide, with substantial social, healthcare, and economic impacts [1,2]. The World Health Organization identifies traffic-related injuries, together with falls, as the leading causes of spinal cord injury [2]. In terms of prevalence, it is estimated that more than 15 million people are living with traumatic spinal cord injury, primarily resulting from road traffic crashes, falls, self-inflicted injuries, sports-related incidents, occupational accidents, and mass-casualty events such as earthquakes or armed conflicts [1,2]. In Europe, in 2023, a total of 900,861 road traffic crashes were recorded, resulting in 20,384 fatalities and 11,143,194 injured individuals [3], highlighting the magnitude of the problem even in settings with advanced road safety systems.

Within the spectrum of trauma-related injuries, road traffic accidents constitute one of the leading causes of acute spinal injury [4]. This anatomical predilection is explained by the biomechanical mechanisms involved in collisions, including rapid acceleration–deceleration, axial compression, and forced flexion–extension movements occurring at the moment of impact [5]. The clinical consequences of these forces range from cervical sprains and stable fractures to complete spinal cord injuries, often resulting in irreversible neurological deficits and imposing a substantial functional and emotional burden on both patients and their families [6].

In this context, the concept of secondary injury is particularly relevant; uncontrolled movement of a previously injured spinal column may induce additional damage, leading to a worsening of the initial pathology [7]. This secondary injury is associated with biomechanical and pathophysiological processes such as further compression of neural tissue, spinal cord ischemia, local inflammation, and impaired perfusion—factors that may exacerbate neurological outcomes even minutes or hours after the initial trauma [8]. Therefore, patient handling, extrication, and early management maneuvers in trauma care play a critical role in preventing this potentially avoidable secondary injury [7,9,10].

Traditionally, cervical immobilization has been considered an essential intervention in patients with suspected spinal cord injury [10], particularly in the prehospital setting. The routine use of rigid cervical collars and immobilization on long spinal boards was incorporated for decades as part of the standard management of trauma patients, with the aim of limiting spinal motion until unstable injuries could be ruled out [11]. However, in recent years, increasing debate has emerged regarding the indiscriminate application of these measures, particularly in conscious, cooperative patients without clear signs of spinal instability [12].

Recent evidence has challenged the traditional assumption that strict cervical motion restriction should remain the primary objective during prehospital extrication. Contemporary consensus statements suggest that, in selected scenarios, reducing entrapment time and expediting transfer to definitive care may provide greater clinical benefit than maximizing spinal motion restriction, particularly when prolonged extrication delays critical interventions [9]. In this context, the EXIT project emphasized that controlled patient movement during rapid extrication may represent a more balanced risk–benefit strategy than prolonged immobilization, especially in unstable or hazardous environments [13].

In parallel, the scientific evidence supporting routine cervical immobilization as an effective measure to prevent secondary neurological injury remains inconclusive. Clinical decision rules, including the NEXUS criteria and the Canadian C-Spine Rule, have demonstrated that selective application of spinal motion restriction can safely reduce unnecessary immobilization in low-risk trauma patients [14,15]. Similarly, the latest Wilderness Medical Society guidelines advocate for an individualized approach based on clinical assessment, patient status, and environmental conditions, rather than the systematic application of universal immobilization protocols [16]. Current recommendations increasingly recognize that many conscious and cooperative patients may not require immobilization devices and identify self-extrication as an appropriate first-line strategy when no contraindications are present. Consequently, contemporary extrication practice increasingly focuses on balancing movement restriction, extrication time, patient condition, and operational circumstances rather than pursuing maximal immobilization in all cases [13].

Available evidence indicates that conventional spinal immobilization may be associated with clinically relevant adverse effects, even in individuals without prior pathology [9,12,17]. Reported effects include alterations in respiratory mechanics, with increased work of breathing and a subjective sensation of dyspnea, as well as the development of tissue ischemia secondary to prolonged pressure application, particularly in the occipital, scapular, and sacral regions [18]. Additionally, increased cervical pain and the onset of headache have been documented, highlighting that these measures, although widely accepted, are not without risks [9,10,17].

This concern is further reinforced by the fact that rigid cervical collar immobilization also complicates airway management, as it restricts mouth opening, reduces neck extension, and impairs glottic visualization during intubation [19,20]. In life-threatening emergency situations, these limitations may translate into an increased number of intubation attempts, longer time to secure the airway, and a higher risk of hypoxia and aspiration [21].

The widely accepted extrication technique in life-threatening emergencies is the Rautek maneuver [22]. It consists of the rapid removal of a victim from a vehicle or confined space using a posterior axillary grip, allowing the rescuer to mobilize the patient without additional equipment. Its main advantage lies in its speed of execution in situations of immediate danger (e.g., fire or risk of explosion), although this rapidity may entail reduced control of spinal alignment and, consequently, a potential increase in vertebral movement [22,23].

**Figure 1:**
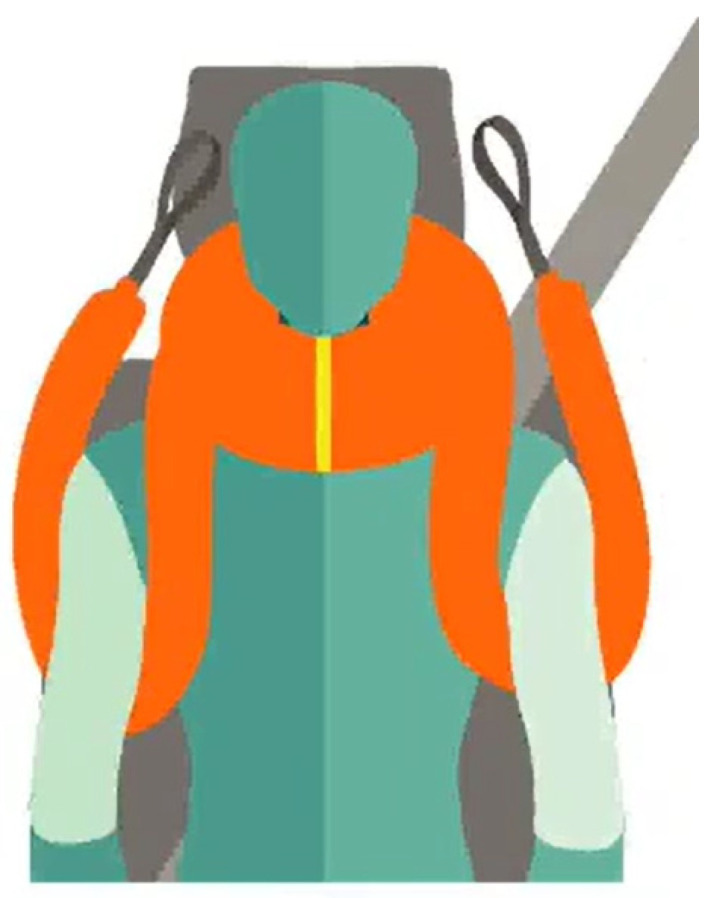
Simulated placement of the SNAID^®^ (source: official SNAID^®^ website).

In this context, the development of devices that combine ergonomics, biomechanics, and ease of use represents a growing trend in emergency medicine [24,25]. The aim of these innovations is to provide effective restriction of spinal motion while minimizing the adverse effects associated with traditional methods [18]. The SNAID^®^ device (Snaid Techologies SL, Córdoba, Spain) is a lightweight, semi-rigid cervical restriction system applied around the neck and upper torso using adjustable anterior and posterior supports that stabilize the cervical segment while leaving the head partially mobile (Figure 1). Its configuration, according to manufacturer information, enables deployment in confined spaces, including vehicle interiors, and its open frontal design facilitates airway access and orotracheal intubation. Within this field, devices such as SNAID^®^ have been introduced and are intended to improve rescuer and patient safety and allow intubation without device removal [26]. These statements derive solely from manufacturer documentation.

Previous studies have already investigated this device. Specifically, Márquez et al. [27], in prior work conducted by the same research group, evaluated its ergonomics, analyzing both the subjective perception of healthcare professionals and the time required for correct application in simulated scenarios, reporting a mean device application time of approximately 40–50 s. In addition, Pons et al. [28] analyzed spinal range of motion and extrication time using inertial sensors in a simulated setting with a healthy subject, providing quantitative data on the limitation of cervical and thoracolumbar movement during rescue maneuvers. Furthermore, Pons et al. [28] compared manual rescue, a rapid extrication device, and an extrication device across 117 firefighters, demonstrating that manual techniques achieved the best alignment and fastest extrication times, while device-assisted methods improved alignment over rapid extrication but prolonged the procedure. These findings are directly relevant to interpreting the potential role of SNAID^®^, which should not be framed as a superior immobilization tool per se, but rather as a device that may offer a compromise between controlled movement and operational efficiency. Both studies provide preliminary evidence regarding the feasibility and potential of the device, although they focus on partial aspects of its use. Therefore, the claim that no comparable devices have been evaluated must be nuanced, acknowledging that existing extrication devices have been studied and that their performance characteristics provide an essential context for interpreting SNAID^®^’s contribution.

To date, no published studies have compared SNAID^®^ with conventional emergency extrication techniques under standardized biomechanical conditions. Therefore, rather than assuming that greater motion restriction necessarily translates into better clinical outcomes, the present study was designed to examine whether the SNAID^®^ system produces different cervical and intersegmental movement patterns than the Rautek maneuver under controlled biomechanical conditions. Accordingly, the general objective of this study was to analyze, using biomechanical sensors, the movements occurring during extrication when using the SNAID^®^ restraint system compared with the Rautek maneuver.

## 2. Materials and Methods

### 2.1. Study Design and Participants

An experimental comparative study was conducted within a controlled simulation environment to quantitatively analyze the biomechanical movements generated in the vertebral column and upper trunk during victim extrication using the SNAID^®^ cervical immobilization system, in comparison with the Rautek maneuver. The study was carried out at the facilities of the University of Almería (Spain) in a standard indoor laboratory environment, ensuring reproducible conditions and environmental control.

A total of 15 volunteer participants took part in the study. Inclusion criteria were (a) enrolment in the Emergency Nursing course within the Bachelor’s Degree in Nursing, and (b) provision of written informed consent. Participants with any musculoskeletal pathology that could interfere with the correct execution of the techniques were excluded.

### 2.2. Data Collection

Each participant performed the victim extrication maneuver under two experimental conditions in the outdoor area of the Faculty of Health Sciences:

(a) Rautek maneuver: a standard mobilization technique in which the rescuer positions their arms under the patient’s axillae, grasps the patient’s forearm, and drags the patient to a safe location.
(b) SNAID^®^ device maneuver: the device is aligned with the patient’s chin; its ends are crossed behind the occiput and securely tightened. The straps are then passed under the patient’s axillae, resting over both clavicles from anterior to posterior. The ends are pulled to extricate and drag the victim to a safe location.

The order of application of both techniques was randomized to minimize learning and fatigue effects. For kinematic data acquisition, four 9-axis inertial measurement units (IMUs; Taobotics, TB-Series Industrial 9-Axis model) were used. Each IMU integrates three sensing subsystems: (1) a triaxial gyroscope for angular velocity estimation; (2) a triaxial accelerometer for linear acceleration measurement; and (3) a triaxial magnetometer for determining absolute orientation relative to the Earth’s magnetic field. The sensor incorporates a proprietary 9-axis magnetic-disturbance-rejection algorithm and a Kalman-filter-based sensor fusion approach, which enables the reconstruction of the full three-dimensional orientation of each instrumented body segment. Additionally, according to the manufacturer, the device achieves approximately 0.1° RMS static orientation accuracy after factory calibration, including compensation for nonlinearity, cross-axis sensitivity, and non-orthogonality effects. 

**Figure 2:**
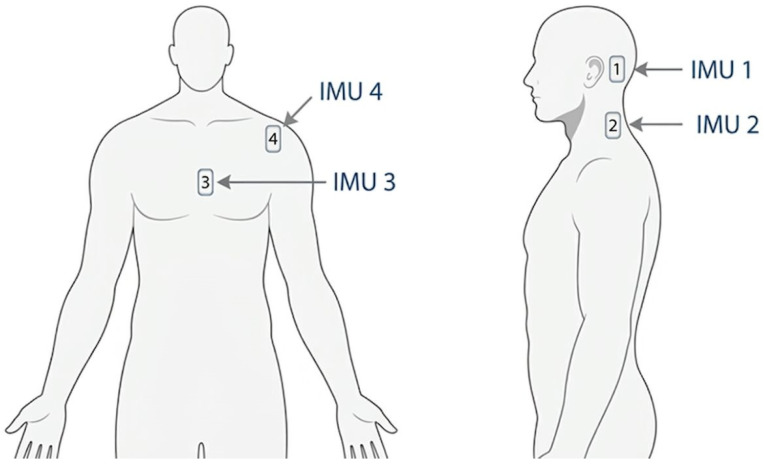
Placement of the IMUs.

The four IMUs were placed at the following anatomical locations:

- IMU 1—Occipital region of the skull (cranial segment).
- IMU 2—Spinous process of the seventh cervical vertebra (C7).
- IMU 3—Sternum (dorsal/trunk segment).
- IMU 4—Left shoulder (Figure 2).

**Figure 3:**
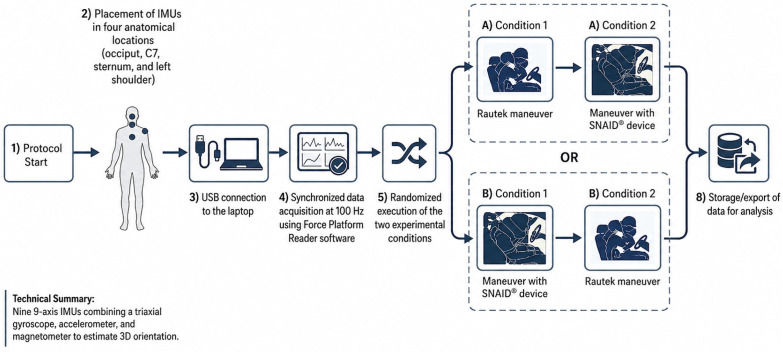
Protocol sequence.

The IMUs were connected via USB interface to a laptop computer. Data acquisition was performed synchronously at a sampling frequency of 100 Hz (sampling period of 10 ms), ensuring sufficient temporal resolution to capture the movements inherent to the extrication maneuvers (Figure 3). Data acquisition, synchronization, and storage were carried out using the open-source software Force Platform Reader (Universidad de Almería, Almería, Spain), developed at the University of Almería [27]. This program enables real-time acquisition of signals from Taobotics IMUs through the Mobile Robot Programming Toolkit (MRPT) library, as well as synchronized management of multiple sensors and export of recorded datasets for subsequent analysis. The software is publicly available at https://aaronpb.github.io/force_platform/ (accessed on 20 June 2026).

A static calibration procedure was performed prior to data acquisition. Specifically, participants adopted a neutral seated posture, which was used as the anatomical reference position for sensor alignment. During this procedure, each IMU was manually aligned with the corresponding body segment. The gravity vector measured by the accelerometer was used to identify the vertical axis, while the remaining axes were defined according to the sensor mounting orientation. This procedure established an initial participant-referenced coordinate frame used for subsequent ROM calculations.

### 2.3. Data Analysis

Statistical analysis of the Range of Motion (ROM) data was carried out using custom Python 3.14 scripts. For each variable and experimental condition, the following statistics were computed: arithmetic mean (M), standard deviation (SD), *p*-value (Wilcoxon Signed-Rank Test) and rank-based effect size (matched-pairs rank-biserial correlation, r_m_^b^), computed as r_m_^b^ = 4(W^+^ − W^−^)/[n(n + 1)]. This effect size is the appropriate companion to the Wilcoxon signed-rank test and is bounded in [−1, +1]. Effect size thresholds: |r_m_^b^| were interpreted as: negligible (<0.10), small (0.10–0.29), medium (0.30–0.49), and large (≥0.50) [29].

To enable inter-trial comparison, all signals were temporally aligned using the global peak (maximum absolute value) of the angular velocity about the trunk’s axis of rotation within the initial phase as a reference event. This point was selected as it corresponds to the onset of the maneuver, defined by the initiation of the subject’s rotation about the trunk axis, and represents a consistent and identifiable feature across trials. This alignment window reflects the portion of the signal selected for kinematic analysis and should not be interpreted as a measured execution time for either maneuver; no timing data were collected in this study, as detailed in the Section Limitations.

Additionally, signals were baseline-corrected by subtracting the reference value at the alignment point, thereby normalizing all measurements to a common zero level. This pro-cedure allows the analysis to focus on relative variations, minimizing inter-trial offsets and improving the robustness of statistical comparisons.

Angular motion for each IMU was decomposed into three axes following Euler angle convention: Lateral Flexion-Extension (ROLL axis), Flexion-Extension (PITCH axis), and Rotation (YAW axis). Relative angular displacements between pairs of adjacent segments (head–trunk and trunk–shoulder) were also calculated as indicators of differential inter-segmental motion.

## 3. Results

From a biomechanical perspective, cervical and intersegmental ROM were analyzed to quantify movement generated during extrication. These measurements provide information regarding movement patterns but should not be interpreted as direct indicators of clinical benefit or neurological protection.

### 3.1. Absolute Angular Displacements

Head segment. The head showed significantly greater ROM with SNAID^®^ in both lateral flexion-extension (*p* = 0.009; r_m_^b^ = 0.75) and sagittal flexion-extension (*p* = 0.023; r_m_^b^ = 0.66). This indicates that the head moves more with SNAID^®^ than with Rautek, which may be explained by the fact that the device restrains the neck but does not fully immobilise the head, allowing a degree of free cephalic motion (*p* = 0.051).

Cervical segment (C7). Lateral flexion-extension of the neck was significantly greater with the Rautek maneuver (*p* = 0.013; r_m_^b^ = −0.71). This result is clinically favourable to SNAID^®^: the device successfully reduces lateral neck movement, which is precisely its protective function. Cervical sagittal flexion-extension and rotation did not show statistically significant differences (*p* = 0.099 and *p* = 0.336, respectively).

Dorsal segment (trunk/sternum). Lateral flexion-extension of the trunk was significantly greater with SNAID^®^ (*p* = 0.003; r_m_^b^ = 0.79), suggesting that restricting the neck may produce compensatory movement in the trunk. Dorsal sagittal flexion-extension and rotation did not show significant differences (*p* = 0.163 and *p* = 0.180).

Shoulder segment. Shoulder lateral flexion-extension was markedly greater with Rautek (*p* < 0.001; r_m_^b^ = −0.99), the largest effect size observed in the entire study, reflecting the axillary grip mechanics inherent to this maneuver. Shoulder rotation was significantly greater with SNAID^®^ (*p* = 0.028; r_m_^b^ = 0.59).

### 3.2. Cumulative Angular Displacement

Head segment. Head flexion-extension showed a large significant difference favouring the SNAID^®^ (r_m_^b^ = +0.65, *p* < 0.001), indicating substantially greater cumulative angular displacement in the sagittal plane. Head rotation was also significant, with a large effect favouring the Rautek technique (r_m_^b^ = −0.37, *p* = 0.041). Lateral flexion-extension did not reach significance and showed only a trivial effect (r_m_^b^ = −0.10, *p* = 0.599).

Cervical segment (C7). Cervical flexion-extension differed significantly between maneuvers, with a large effect favouring the Rautek technique (r_m_^b^ = −0.53, *p* = 0.002), indicating greater cumulative cervical demand in the sagittal plane with that technique. Lateral flexion-extension and rotation showed no significant differences, with trivial-to-small effects (r_m_^b^ = −0.02 and +0.07, respectively; *p* = 0.885 and *p* = 0.676).

Dorsal segment. Dorsal flexion-extension was the only significant variable in this segment, with a large effect favouring the SNAID^®^ (r_m_^b^ = +0.43, *p* = 0.030). Lateral flexion-extension and rotation showed medium-sized trends in opposite directions, but neither reached significance (*p* = 0.117 and *p* = 0.115, respectively).

Shoulder segment. Shoulder lateral flexion-extension showed the largest effect across the entire cumulative displacement analysis, with a very large difference favouring the Rautek technique (r_m_^b^ = −0.79, *p* < 0.001), indicating markedly greater cumulative displacement in the frontal plane with that maneuver. Shoulder rotation was also significant, with a large effect favouring the SNAID^®^ (r_m_^b^ = +0.37, *p* = 0.031). Flexion-extension did not reach significance (r_m_^b^ = −0.30, *p* = 0.208).

### 3.3. Relative Angular Displacements Between Segments

Table 1 shows the median [IQR] values of the joint angles (IMU_ANG) recorded for each mobilization technique, along with the results of the comparison using the Wilcoxon Signed-Rank test. Statistically significant differences between the two techniques were observed for Head Lateral Flexion-Extension (*p* < 0.001), Head Flexion-Extension (*p* = 0.001), Dorsal Lateral Flexion-Extension (*p* < 0.001), Shoulder Flexion-Extension (*p* = 0.033) and Shoulder Rotation (*p* = 0.007).

## 4. Discussion

The objective of this study was to analyze, using biomechanical sensors, the movements occurring during extrication when using the SNAID^®^ restraint system compared with the Rautek maneuver. The results show relevant differences between both techniques, with direct biomechanical and clinical implications, not only in terms of statistical significance but also in the magnitude of the observed effects, several of which were large effect (|r_m_^b^| > 0.50), reinforcing the magnitude and consistency of the observed biomechanical differences.

First, regarding head movement, the SNAID^®^ device showed higher values in flexion–extension. This phenomenon may be explained by selective cervical stabilization without complete head fixation, a behaviour described in studies on partial immobilization and motion restriction [17,28]. Research on immobilization systems has suggested that complete restriction of all spinal segments is not always achievable or necessary, provided that the cervical segment is adequately protected [22,30]. In addition, this approach may facilitate airway opening for oropharyngeal airway placement with the SNAID^®^ device in situ, without requiring its removal, and may even allow positioning of the patient in the lateral safety position while maintaining cervical motion coupling and avoiding cervical step-off. It is noteworthy that cephalic rotation did not reach statistical significance, suggesting that the increase in movement with SNAID^®^ is mainly concentrated in flexion–extension planes. Complementarily, the cumulative angular displacement analysis confirmed this pattern: SNAID^®^ generated significantly greater accumulated head flexion–extension across the entire maneuver, whereas peak ROM values alone may underestimate the total cephalic excursion occurring during a dynamic extrication task. This finding reinforces the interpretation that the device constrains the cervical segment while permitting a degree of rhythmic cephalic oscillation throughout the procedure.

Regarding the neck (C7), no significant differences were observed in sagittal flexion–extension or rotation. The significant reduction in lateral flexion–extension with the SNAID^®^ device constitutes one of the most relevant findings. Previous studies have shown that even small displacements in the cervical spine may be clinically relevant in patients with instability [31]. In our study, the Rautek maneuver generated greater cervical lateral displacement, indicating that SNAID^®^ effectively reduces movement in this critical segment. However, these findings should be interpreted cautiously, as reduced intersegmental cervical displacement does not necessarily imply lower absolute movement of the head–neck complex as a whole. Rather than demonstrating complete immobilization, the data suggest that SNAID^®^ may promote a more coupled movement pattern between the head and trunk during extrication. In this sense, the lower mobility observed with SNAID^®^ indicates that the device modifies cervical movement patterns during extrication [9,11]. It should be noted, however, that peak ROM and cumulative angular displacement capture complementary aspects of movement: whereas lateral flexion–extension ROM favoured SNAID^®^, the cumulative displacement analysis revealed that Rautek generated significantly greater accumulated cervical sagittal displacement across the maneuver. These two metrics are not contradictory but reflect different dimensions of spinal loading, and both should be considered when evaluating cervical protection during extrication.

On the other hand, regarding the trunk, the increase in lateral movement with the SNAID^®^ device could reflect a biomechanical compensatory mechanism. This type of movement redistribution has been described in spinal biomechanics studies, where restriction in one segment induces greater mobility in adjacent segments [32]. Specifically, the trunk showed greater lateral flexion–extension with SNAID^®^, while no significant differences were found in other planes, reinforcing the hypothesis of localized compensation. Nevertheless, an alternative explanation should also be considered. Because the SNAID^®^ system applies traction through the head, shoulders, and upper thorax, part of the observed increase in absolute head and trunk motion may reflect the participant being mobilized as a coupled unit during extraction rather than isolated cervical spine movement itself. The very low relative head-to-trunk displacement observed with SNAID^®^ supports this possibility. Therefore, although the intersegmental findings are compatible with a compensatory redistribution mechanism, the present design does not allow definitive differentiation between true compensatory segmental motion and coupled whole-body displacement generated by the extraction mechanics of the device. From a clinical perspective, the increased lateral trunk movement with SNAID^®^ should therefore be interpreted cautiously, as it may represent either compensatory motion secondary to reduced cervical mobility or a consequence of coordinated movement of the head–trunk complex during extrication. The cumulative angular displacement data partially support this interpretation: dorsal flexion–extension accumulated displacement was significantly greater with SNAID^®^, consistent with a compensatory sagittal redistribution pattern. However, lateral flexion–extension did not reach significance in the cumulative metric, suggesting that the compensatory effect is primarily expressed in the sagittal plane and that caution is warranted when generalising across movement axes. This interpretation is supported by the intersegmental findings observed in the present study. Although greater absolute motion of the head and trunk was recorded with SNAID^®^, head–trunk intersegmental displacement was substantially lower than with the Rautek maneuver. If the increased absolute motion primarily reflected compensatory movement resulting from restriction at the cervical level, larger relative displacements between the head and trunk would be expected. Instead, the low intersegmental values suggest that these segments moved more synchronously during extraction, supporting the hypothesis of a more coupled head–neck–trunk movement pattern. Nevertheless, because the present study was limited to external kinematic measurements, the underlying biomechanical mechanisms cannot be established definitively and should be explored in future investigations.

Regarding the shoulder, the most notable finding was observed at this level, where the Rautek maneuver generated a very pronounced increase in lateral movement. This result is consistent with the mechanics described for this maneuver, which is widely used in rescue situations but is not specifically designed to limit spinal motion [30]. In absolute terms, this difference was particularly marked, representing the largest effect size in the entire study. Although shoulder movement has a less direct impact on the spinal cord, it may indicate reduced overall stability during patient handling. Conversely, shoulder rotation was greater with the SNAID^®^ device, which could reflect a redistribution of movement toward more peripheral and less critical segments. The cumulative displacement analysis further substantiates this finding: shoulder lateral flexion–extension showed the largest effect size, confirming that the Rautek axillary grip generates not only greater peak displacements but also substantially higher total accumulated frontal-plane shoulder motion throughout the maneuver. This convergence between peak ROM and cumulative metrics strengthens the clinical interpretation that the Rautek technique imposes markedly greater mechanical demand on the shoulder girdle.

Considering relative movements, the analysis of intersegmental desynchronization allows identification of how differential displacements between the head and trunk may promote tensile or shear loading of the spine when adequate coupling is not maintained [32,33]. In this regard, the results show particularly relevant differences, with markedly higher values in the Rautek maneuver in both lateral and sagittal flexion–extension, indicating clear intersegmental desynchronization. In our data, the SNAID^®^ device demonstrates greater coherence in coupled movement, thereby reducing the magnitude of these displacements, a behaviour consistent with cervical load models [33]. This pattern is also observed in the trunk–shoulder complex, where all analyzed variables were significantly higher with the Rautek maneuver, suggesting lower overall mechanical stability during the procedure. Overall, the observed pattern suggests that SNAID^®^ concentrates movement in less critical regions, whereas the Rautek maneuver produces more pronounced cervical mobilization, a particularly relevant aspect in scenarios where reduction in cervical motion is considered clinically appropiate [31].

Beyond the biomechanical findings, some operational considerations may also be relevant. The use of a structured extrication device such as SNAID^®^ could theoretically reduce direct physical contact between rescuer and patient and promote more ergonomically favorable handling positions compared with manual techniques such as the Rautek maneuver. Previous literature on patient-handling devices suggests potential benefits regarding biosafety and musculoskeletal load reduction for emergency personnel [34,35,36,37,38,39]. However, these aspects were not directly evaluated in the present study and therefore should be interpreted only as inferential considerations rather than demonstrated advantages of the device. Future studies incorporating workload analysis, ergonomic assessment, and operational performance metrics will be necessary to determine whether these theoretical benefits translate into measurable clinical or occupational improvements.

The present findings must also be situated within the current international guideline context, which has shifted substantially away from treating absolute movement minimisation as the central goal of extrication practice. The 2025 FPHC consensus statement [40] establishes as its first and primary recommendation that all injured patients should be considered time-dependent, with the primary focus of the extrication plan being minimisation of entrapment time rather than maximisation of spinal restriction. The 2025 NAEMSP comprehensive review [9], concluded that there are no published data supporting spinal immobilisation or spinal motion restriction as standard of care and that efforts to reduce cervical collar use should be considered. The present kinematic findings should not be interpreted as evidence that a cervical restraint device of this kind ought to be adopted; rather, they indicate that SNAID^®^ measurably alters cervical and intersegmental movement patterns relative to the Rautek maneuver under standardised simulation conditions, a finding whose clinical weight is necessarily limited by the absence of patient outcome data, by the absence of extrication time data discussed above, and by the fact that the Rautek maneuver itself is a technique designed for immediate-danger scenarios in which speed, not motion restriction, is the explicit priority. In addition, the FPHC consensus statement identifies self-extrication as the preferred approach for many alert patients without contraindications. Consequently, the present comparison between SNAID^®^ and the Rautek maneuver should not be interpreted as representing the full spectrum of contemporary extrication strategies. Rather, it evaluates the relative biomechanical characteristics of two assisted extraction approaches under standardized conditions.

The present study should be interpreted as a preliminary biomechanical analysis aimed at comparing the relative motion patterns generated by two different extrication techniques under controlled conditions, rather than as a direct replication of real-world trauma scenarios. Although the participants were healthy volunteers, the standardized simulation model enabled consistent acquisition of kinematic data while minimizing potential confounding factors.

### Limitations

This study presents several limitations that should be considered when interpreting the findings. First, the inclusion of healthy nursing students instead of injured patients or professional rescue personnel limits the representativeness of the findings for real prehospital trauma scenarios. Biomechanical responses during actual rescue situations may differ due to factors such as pain, cervical instability, emotional stress, muscle guarding, body habitus, or environmental constraints. Therefore, the present findings should be interpreted as exploratory biomechanical evidence obtained under standardized simulation conditions rather than as definitive clinical evidence. Furthermore, the analysis was limited to kinematic parameters, without incorporating kinetic data, muscular activation patterns, or operational variables such as extrication time, rescuer workload, or patient-reported outcomes, which restricts a more comprehensive assessment of clinical performance. Critically, no extrication time data were collected for either condition in this study, and this is a substantive limitation. Current international guidance identifies minimisation of entrapment time as the primary clinical priority during extrication, meaning that the time cost associated with any restraint device is not a secondary consideration but a central determinant of its clinical utility. Consequently, the biomechanical findings reported should be interpreted as evidence that SNAID^®^ modifies cervical and intersegmental movement patterns under standardised conditions, rather than as evidence that the device offers a net clinical or operational advantage once time cost is taken into account. Another limitation is the absence of a self-extrication comparison group. Current international guidance recognizes self-extrication as a preferred strategy for many conscious patients. Consequently, the present findings are limited to the comparison of two assisted extrication techniques and cannot be generalized to all contemporary extrication pathways. Future research should therefore focus on validation in real-world prehospital and emergency settings, including studies with experienced professionals and larger multicenter samples. Additionally, integrating kinetic measurements, biomechanical modeling, workload assessment tools, and standardized extrication time analysis would provide a more holistic understanding of device performance. Long-term investigations should also explore patient-centered outcomes, including neurological sequelae, respiratory impact, and pain, to determine the true clinical effectiveness and safety profile of the SNAID^®^ system compared with conventional extrication techniques.

## 5. Conclusions

The findings obtained in the present study suggest that the SNAID^®^ device produced different biomechanical movement patterns compared with the Rautek maneuver during the extrication of patients with suspected cervical spine injury. In particular, the significant reduction in cervical spine movement in the lateral plane and the lower intersegmental desynchronization (especially in the head–trunk complex, with differences reaching up to 40° in sagittal flexion–extension) reinforce its ability to reduce intersegmental displacement. These findings may be relevant in the context of spinal motion restriction, although their direct clinical implications cannot be established from the present biomechanical model alone.

The SNAID^®^ device was associated with lower relative cervical movement compared with the Rautek maneuver, while the overall movement pattern observed suggests greater mechanical cohesion between body segments. These findings indicate differences in biomechanical behavior between the two techniques under standardized simulation conditions; however, because neurological outcomes, injury progression, patient safety endpoints, and operational performance were not evaluated, the clinical significance of these differences cannot be determined. Similarly, although the Rautek maneuver generated greater relative intersegmental mobility, which could theoretically increase shear-type displacement across the spine, the present data do not allow conclusions regarding clinical superiority or patient benefit.

The head fixation provided by the SNAID device is not absolute, which reflects a functional design intended to balance restriction of the head–neck axis with airway management requirements. This feature allows cervical alignment to be maintained while enabling airway access when clinically necessary. In this regard, although higher head mobility values were observed with SNAID^®^, these may reflect coupled movement of the head–trunk complex during extraction rather than isolated cervical mobilization. The controlled range of head movement may facilitate airway management maneuvers without requiring device removal or additional head–neck manipulation. Nevertheless, these potential operational implications were not directly assessed in the present study and should therefore be interpreted cautiously.

Overall, the findings of this study support further evaluation of SNAID^®^ as an alternative extrication approach capable of modifying cervical and intersegmental movement patterns when compared with the Rautek maneuver under standardized simulated conditions. Further research incorporating real-world emergency scenarios, extrication time, ergonomic assessment, and patient-centered clinical outcomes will be necessary to determine the practical and clinical relevance of these biomechanical findings.

## Figures and Tables

**Table 1:** Relative ROM between adjacent segments.

Variable	Median SNAID (º)	[IQR] SNAID	Median Rautek (º)	[IQR] Rautek	*p*-Value	r_m_^b^
Head Lateral Flexion-Extension	32.99	10.98	21.23	10.85	<0.001	−0.92
Head Flexion-Extension	40.55	11.99	34.06	4.36	<0.001	−0.99
Head Rotation	132.95	18.72	140.88	9.53	0.787	−0.07
Dorsal Lateral Flexion-Extension	23.78	6.88	14.43	8.19	<0.001	−0.94
Shoulder Lateral Flexion-Extension	19.57	10.89	57.69	6.99	0.033	−0.55
Shoulder Rotation	142.36	11.35	129.25	4.25	0.007	−0.77

Values are expressed as median [interquartile range, IQR = Q3 − Q1], in degrees (°). A *p*-value < 0.05 was considered statistically significant (Wilcoxon Signed-Rank test).

## Data Availability

Dataset available on request from the authors.

## Abbreviations

The following abbreviations are used in this manuscript:

IMUs	Inertial Measurement Units
